# Characterization of T Lymphocytes in Chronic Obstructive Pulmonary Disease

**DOI:** 10.1371/journal.pmed.0010020

**Published:** 2004-10-19

**Authors:** Peter J Barnes, Manuel G Cosio

## Abstract

A new study adds to the mounting evidence implicating T cells as an important component of the inflammation in chronic obstructive pulmonary disease

Chronic obstructive pulmonary disease (COPD) is a global epidemic of major proportions that is predicted to become the third most common cause of death and fifth most frequent cause of chronic disability by 2020. In developed countries it is mainly caused by cigarette smoking, but the reasons why only a proportion (10%–20%) of smokers develop progressive airflow limitation is currently unknown. The disease is characterized by a chronic inflammatory process predominantly in the small airways and lung parenchyma, with increased numbers of macrophages, neutrophils, and T lymphocytes [1]. The difference between smokers without COPD and smokers with COPD appears to be the intensity rather than the nature of the inflammatory process. This inflammation in the small airways is associated with fibrosis and increases with the severity of airflow limitation [2], which has led to the view that COPD represents an amplification of the normal inflammatory response to inhaled irritants such as cigarette smoke.

## T Lymphocytes in COPD

T lymphocytes were first reported to be increased in patients with COPD by Finkelstein and colleagues, who showed a correlation between the number of T lymphocytes/mm^3^ of lung and the extent of emphysema [3]. It was later shown that both CD4^+^ (T helper) and CD8^+^ (suppressor/cytotoxic) T cells were increased in the airways and lung parenchyma of patients with COPD, with a predominance of CD8^+^ cells [4,5]. This is in contrast to the findings in asthma, in which there is a predominance of CD4^+^ cells, which are predominantly of the T helper 2 (Th2) pattern, with increased expression of interleukin (IL)-4, IL-5, and IL-13 (see Glossary), and which are associated with an increased number of eosinophils. In smokers who develop COPD there appears to be activation of adaptive immunity, with the infiltration of CD8^+^ and CD4^+^ cells in the alveolar walls and small airways and—in patients with the most severe disease—the presence of lymphoid follicles that contain a core of B lymphocytes surrounded by T cells [2]. This activation presumably follows on from the initial and then sustained innate immune response characterized by increased numbers of macrophages and neutrophils; it may involve the migration of dendritic cells from the epithelium to the local lymph nodes and presentation of antigenic substances to T cells, resulting in clonal expansion of CD4^+^ and, to an even greater extent, CD8^+^ cells.

The study by Grumelli et al. (2004) published in this issue of *PLoS Medicine* takes the story forward [6]. The CD4^+^ and CD8^+^ cells appear to be fully activated, as they would be after being presented with antigens, and they show predominantly a T helper 1 (Th1)/cytotoxic T 1 (Tc1) pattern, with increased expression of interferon-γ (IFN-γ) and Th1 chemokines. This is consistent with the recent demonstration of increased expression of IL-12 in bronchial biopsies of patients with COPD and activation of the transcription factor STAT-4 in T cells, subsequent STAT-4 nuclear translocation, and IFN-γ gene induction, and thus a Th1 commitment in the T cells [7].

**Figure 1:**
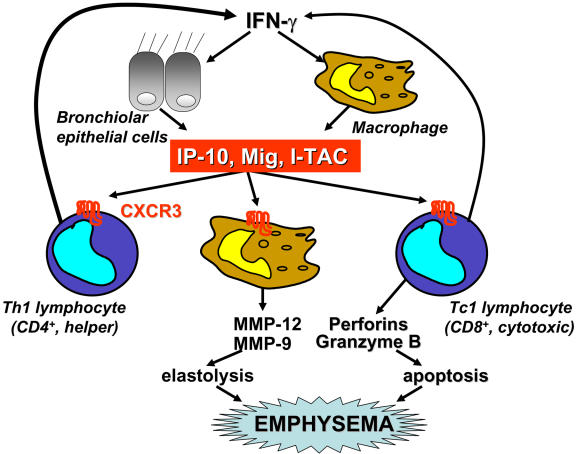
In Emphysema, a Self-Perpetuating Loop May Lead to Accumulation of Activated Th1/Tc1T Cells in the Peripheral Lung

As well as producing the cytokines IL-2 and IFN-γ, Th1 and Tc1 cells also express the chemokine receptor CXCR3 and the ligands that activate this receptor, IFN-γ inducible protein 10 (IP-10, CXCL10), monokine induced by IFN-γ (CXCL9), and IFN-inducible T cell α chemoattractant (CXCL11). There is an increase in the expression of IP-10 in the airways of patients with COPD and an increase in the number of CXCR3^+^ cells [8]. CXCR3 is expressed on Th1/Tc1 cells, macrophages, and epithelial cells. Release of CXCR3-activating chemokines would attract Th1 and Tc1 cells into the lungs, and these cells then release IFN-γ, which releases more CXCR3 chemoattractants. This results in a self-perpetuating loop that may lead to accumulation of activated Th1 and Tc1 cells in the peripheral lung (Figure 1).

## Role of Cytotoxic T Cells

It is likely that Th1 cells are the major source of IFN-γ in the lungs of patients with COPD and therefore drive and maintain the T cell response and promote an “immune inflammation” with neutrophils and macrophages. However, it is the role of Tc1 cells that is of particular interest, as these cells are cytotoxic to epithelial cells through the release of granzymes and perforins, which induce apoptosis. Increased concentrations of perforins have recently been reported in the sputum of patients with COPD [9]. In support of this idea there is an increase in the apoptosis of alveolar cells in the lungs of patients with COPD, and this is correlated with the number of CD8^+^ cells and the severity of emphysema [10].

## T Cell Perpetuation

The T cell inflammatory response appears in mild COPD but increases markedly with disease severity. It is possible that the initial immune response becomes self-perpetuating because of endogenous autoantigens resulting from inflammatory and oxidative lung injury. There are also antigens in tobacco, but the inflammatory response appears to become independent of smoking status, and there is intense inflammation even in patients who stopped smoking many years previously [2], as seen in the present study by Grumelli et al. [6]. Another possibility is that this chronic immune response is driven, or at least maintained, by chronic infection of the respiratory tract often seen in patients with severe disease, in which there is increased colonization of the lower airways. These infections could act as co-stimulators, or by antigenic mimicry or as polyclonal activators they could provide a persisting antigenic stimulus and maintain the inflammatory process. Further studies on T cell receptor usage and expression of surface markers may give further clues as to the driving mechanisms for the increased Th1 and Tc1 cells in COPD.

## Proteases

COPD is characterized by destruction of the lung parenchyma and loss of elastin due to elastolytic enzymes, such as neutrophil elastase and certain matrix metalloproteinases (MMPs). The predominant MMP in COPD appears to be MMP9, which is released in much larger amounts from alveolar macrophages of patients with COPD than from those of smokers without the disease [11]. The study by Grumelli et al. showed that CXCR3 ligands led to the expression of the elastolytic enzyme MMP12 in alveolar macrophages and that this process was increased in the lungs of patients with COPD. This finding provides a neat link between T cells and alveolar destruction, but is discrepant with other data that have failed to show significant MMP12 release from macrophages of patients with COPD [11].

## Therapeutic Implications

There are currently no treatments that reduce the relentless progression of COPD, and none that have significant anti-inflammatory effects. However the recognition that an adaptive immune T cell response, most likely driven by antigens, may play an important pathophysiological role in the pathogenesis of COPD has important therapeutic implications. It is possible that T cell inhibitory strategies, such as the use of immunosuppressants, might be effective, although side effects may be a problem, and there is particular concern about increasing the risk of bacterial infection. Another approach might be to block the trafficking of Th1 and Tc1 cells to the lungs by blocking CXCR3, and there is now a search for small-molecule inhibitors of these receptors. Inhibition of IFN-γ signaling might be another approach.

The mounting evidence implicating T cells, and thus an adaptive immune response, as an important component of the inflammation in COPD is overwhelming. A better understanding of the immune mechanisms involved in COPD is important, since it might lead us to new and more effective therapeutic approaches to this important disease.

**Glossary**

**CD4^+^ (helper) T cell:** T lymphocyte that enhances the inflammatory response

**CD8^+^ (cytotoxic/suppressor) T cell:** T lymphocyte that suppresses the inflammatory response

**CXCR3:** Chemokine receptor that is selectively activated by IP-10, monokine induced by IFN-γ, and IFN-inducible T cell chemoattractant

**Cytotoxic (Tc1) cell:** T cell that is characterized by secretion of INF-γ

**Granzyme:** Enzyme released by cytotoxic T cells

**Interferon-γ inducible protein 10 (IP-10, CXCL10):** Chemokine of 10 kDa that selectively activates CXCR3

**Interferon-inducible T cell γ chemoattractant (I-TAC, CXCL11):** Chemokine that selectively activates CXCR3

**Interferon-γ (IFN-γ):** Protein secreted by Th1 and Tc1 cells

**Interleukin-4 (IL-4):** Protein secreted by Th2 cells that is important in increasing IgE secretion

**Interleukin-5 (IL-5):** Protein secreted by Th2 cells that is important for eosinophilia

**Interleukin-12 (IL-12):** Protein secreted by antigen-presenting cells that promotes differentiation of Th1 cells

**Interleukin-13 (IL-13):** Protein secreted by Th2 cells that is important for IgE secretion

**Matrix metalloproteinase (MMP):** Proteolytic enzyme that degrades connective tissue

**MMP9, MMP12:** MMPs that destroy elastin fibers

**Monokine induced by interferon-γ (MIG, CXCL9):** Chemokine that selectively activates CXCR3

**Neutrophil elastase:** Enzyme released from neutrophils that destroys elastin fibers

**Perforin:** Protein released by cytotoxic T cells that induces apoptosis

**STAT-4:** Transcription factor specifically activated by IL-1

**T helper 1 (Th1) cell:** T lymphocyte that is characterized by secretion of INF-γ

**T helper (Th2) cell:** T lymphocyte that is characterized by increased secretion of the cytokines IL-4, IL-5, and IL-13; characteristically increased in allergic inflammation

## Abbreviations

COPD: chronic obstructive pulmonary disease
IFN-γ: interferon-γ
IL: interleukin
IP-10: interferon-γ inducible protein 10
MMP: matrix metal-loproteinase
Tc1: cytotoxic T 1
Th1: T helper 1
Th2: T helper 2
